# Supplementary material for: MetaRibo-Seq measures translation in microbiomes
Source: Nat Commun. 2020 Jun 29;11:3268. doi: 10.1038/s41467-020-17081-z (PMC7324362; doi:10.1038/s41467-020-17081-z)
Supplement: Supplementary file 10 — Supplementary Data 7 [file 41467_2020_17081_MOESM10_ESM.zip › File2/Confidence_High_Taxonomy/84751_out.krona.html]

Javascript must be enabled to view this page.

members
magnitude
magnitudeUnassigned
count
unassigned
taxon
rank

84751\_out

8


SRS024388\_contig\_number\_contig-100\_14887.14887SRS104693\_contig\_number\_contig-100\_22846.70617
8
2
superkingdom
2

phylum
1239
6

class
6
186801

6
186802
order

541000
6
family

1
genus
1263
6

SRS047044\_contig\_number\_contig-100\_11473.53442

5
41978

SRS013687\_contig\_number\_4650SRS018656\_contig\_number\_contig-100\_2897.41328SRS019968\_contig\_number\_contig-100\_1878.90325SRS075078\_contig\_number\_contig-100\_15688.52018SRS150029\_contig\_number\_11381
species
